# Increased Neuronal α-Synuclein Pathology Associates with Its Accumulation in Oligodendrocytes in Mice Modeling α-Synucleinopathies

**DOI:** 10.1371/journal.pone.0046817

**Published:** 2012-10-15

**Authors:** Haya Kisos, Katharina Pukaß, Tamir Ben-Hur, Christiane Richter-Landsberg, Ronit Sharon

**Affiliations:** 1 Biochemistry and Molecular Biology, IMRIC, The Hebrew University-Hadassah Medical School, Jerusalem, Israel; 2 Biology, Molecular Neurobiology, University of Oldenburg, Oldenburg, Germany; 3 Neurology, Hadassah University Medical Center, Jerusalem, Israel; National Institutes of Health, United States of America

## Abstract

Multiple system atrophy (MSA) is a progressive neurodegenerative disorder characterized by striatonigral degeneration and olivo-pontocerebellar atrophy. The histopathological hallmark of MSA is glial cytoplasmic inclusions (GCI) within oligodendrocytes, accompanied by neuronal degeneration. MSA is a synucleinopathy, and α-Synuclein (α-Syn) is the major protein constituent of the GCI. It is unclear how the neuronal α-Syn protein accumulates in oligodendrocytes. We tested the hypothesis that oligodendrocytes can take up neuronal-secreted α-Syn as part of the pathogenic mechanisms leading to MSA. We report that increases in the degree of α-Syn soluble oligomers or intracellular α-Syn levels, enhance its secretion from cultured MN9D dopaminergic cells, stably expressing the protein. In accord, we show that primary oligodendrocytes from rat brain and oligodendroglial cell lines take-up neuronal-secreted or exogenously added α-Syn from their conditioning medium. This uptake is concentration-, time-, and clathrin-dependent. Utilizing the demonstrated effect of polyunsaturated fatty acids (PUFA) to enhance α-Syn neuropathology, we show an *in vivo* effect for brain docosahexaenoic acid (DHA) levels on α-Syn localization to oligodendrocytes in brains of a mouse model for synucleinopathies, expressing human A53T α-Syn cDNA under the PrP promoter. Hence, pathogenic mechanisms leading to elevated levels of α-Syn in neurons underlie neuronal secretion and subsequent uptake of α-Syn by oligodendrocytes in MSA.

## Introduction

Multiple system atrophy (MSA) is an adult-onset, progressive, neurodegenerative disorder with clinical symptoms of autonomic failure and motor impairment. The pathological hallmark of MSA is glial cytoplasmic inclusions (GCI) in oligodendrocytes, with α-Synuclein (α-Syn) as their major protein constituent [1], [2]. Evidence from genetic studies supports a role for α-Syn in MSA. Specifically, analysis of single nucleotide polymorphisms (SNPs) in the α-Syn gene has identified an association between certain α-Syn SNPs and an increased risk for the development of MSA [3], [4].

While the mechanisms of neurodegeneration in MSA remain unclear, a common hypothesis is that oligodendrocytes degenerate as a result of accumulation and deposition of α-Syn within their cytoplasm (recently reviewed in [5], [6]). A growing body of evidence suggests that α-Syn is released from healthy neurons. Specifically, a small portion of α-Syn is released via exocytic vesicles [7], [8]. The secretion process may also occur with misfolded, cytotoxic forms of α-Syn, thereby allowing the “spread” of abnormal α-Syn to neighboring cells [9], [10], [11], [12], [13]. α-Syn is detected in cerebrospinal fluid (CSF) [14], plasma [15], red blood cells [16], [17] and skin fibroblasts [18]. While it is currently unclear whether neuronal secreted α-Syn may be used to discriminate healthy and PD-patients, the occurrence of α-Syn in blood and plasma may potentially explain its presence and toxicity in melanomas [19], [20] and the epidemiological findings suggesting increased incidents of melanoma among people with PD [21].

Neuronal-secreted α-Syn could potentially underlie the pathogenic mechanisms in MSA. Although α-Syn mRNA and protein were detected in rat brain oligodendrocytes [22], no evidence for α-Syn expression was found in oligodendroglia from healthy and MSA human brains [23]. Therefore, neuronal secreted α-Syn protein could contribute or initiate the cytoplasmic inclusions routinely found in oligodendrocytes in MSA.

Recently, we have reported that the degree of neuronal α-Syn pathology in A53T α-Syn tg mice can be controlled by dietary alterations in brain docosahexaenoic acid (DHA) levels [24]. Specifically, a diet enriched in DHA increased the accumulation of soluble and insoluble neuronal α-Syn. In accord with cytotoxic α-Syn accumulations, we detected evidence for enhanced neuritic injury and astrocytosis. Conversely, α-Syn deleterious effects were significantly attenuated by a low-DHA (and low n-3 PUFA) diet.

The present study was undertaken to investigate the mechanisms involved in α-Syn accumulation in oligodendrocytes. We demonstrate that dopaminergic cells, stably overexpressing α-Syn, release the protein into their growth medium. Intracellular levels of total α-Syn and levels of soluble α-Syn oligomers, two parameters that strongly relate to α-Syn toxicity, positively correlate with the secretion. Importantly, we show that α-Syn secretion is a specific mechanism, which does not occur for the β-Synuclein (β-Syn) protein homolog. The data further demonstrate that neuronal-secreted or exogenously added purified α-Syn is internalized into cells of oligodendroglial origin in a clathrin-dependent manner. Utilizing A53T α-Syn transgenic mice, we further show that enhances in α-Syn neuropathology, obtained by alterations in dietary brain DHA levels, modulate the degree of α-Syn protein detected in oligodendrocytes. Based on these results we suggest, that neuronal-secreted α-Syn may play a role in the pathogenic accumulations of α-Syn within oligodendrocytes, as observed in MSA.

## Materials and Methods

### Animals and diets

The A53T α-Syn tg mouse line [25] was purchased from Jackson laboratories (Bar Harbor Maine USA) and bred to homozygousity. Similar to the original description, the mice remained healthy up to the age of ∼7 months. At 8–9 months of age, the colony began to develop motor and behavior phenotype as described previously [24]. In parallel, we maintained a non-transgenic (ntg) mouse (C57Bl/6) (Jackson Laboratories, Bar Harbor Maine USA) and an α-Syn −/− [26] colonies. This study was carried out in strict accordance with the recommendations in the Guide for the Care and Use of Laboratory Animals of the National Institutes of Health. The protocol was approved by the Authority of Biological and Biomedical Models of the Hebrew University of Jerusalem, NIH approval # OPRR-A01-5011 (Permit Number: MD-09-12084).

Diet administration was as described [24]. Briefly, males and females (n = 10–15) of A53T α-Syn and age-matched ntg mice (B6) were randomly assigned into three groups and were fed for 230 days either one of the three diets, a standard mouse chow (2018SC+F, Harlan Teklad, Madison, WI); a safflower-based diet depleted of DHA, low-DHA diet (TD 00522, Harlan Teklad, Madison, WI); or the above low DHA, (and low n-3 PUFA diet) supplemented with 0.69% w/w DHA (Martek Bioscience, Columbia, MD) (TD 07708, high DHA diet) [27], [28]. The contents of total fat (6%); proteins (18%) and carbohydrate (49%) are identical between the diets. At termination of the experiment, mice were anesthetized with an intraperitoneal overdose injection of sodium pentobarbitone (1 ml/1.5 kg), and were then perfused with PBS buffered formalin. Brains were removed and fixed for another 24 hours in formalin.

### Cell cultures

The mesencephalic neuronal cell line, MN9D [29], with dopaminergic properties was transfected with wt or A53T human α-Syn or β-Syn cDNA in the pCDNA 3.1 vector. Since α-Syn expression and oligomerization are dynamic in these clones [30], comparisons were made between clones of wt and A53T that were maintained in parallel from DNA transfection and selection of stable clones to the actual measurements [30]. Oli-neu [31] and OLN93 [32] cell lines, derived from oligodendroglial origin, were cultured according to their original description.

*Primary Oligodendrocytes* were prepared as previously described [32]. Briefly, primary cultures of glial cells were prepared from brains of 1–2 day old Wistar rats sacrificed by decapitation. Oligodendrocytes were mechanically removed after 10–14 days in culture by shaking the flasks on an orbital lab-shaker (New Brunswick Scientific, Edison, NJ, USA). Oligodendrocyte precursor cells were re-plated on poly-L Lysine (PLL)-coated culture dishes (2.7×10^6^ cells/10 cm dish) supplemented with glass cover slips (Fisher scientific, Schwerte, Germany). Cells were grown in DMEM with 0.5% heat-inactivated foetal calf serum (FCS) supplemented with insulin (5 µg/ml), transferrin (5 µg/ml), and sodium selenite (5 ng/ml) (Roche Diagnostics, Mannheim, Germany) in a 10% CO_2_-atmosphere for 5 days. These cultures contain a highly enriched population of differentiated oligodendrocytes with mature morphology.

### α-Syn uptake by cultured cells

Cells were plated on cover slips pretreated with PLL (1 mg/ml or 50 µg/ml for Oli-neu and OLN93 cells, respectively). Following incubation for 24 hours in standard growth medium, cells were incubated for 16 hours longer in medium supplemented with purified human recombinant α-Syn protein. Alternatively, Oli-neu or OLN93 cells were incubated in conditioned medium (CM) collected from MN9D cell (naïve or over expressing α-Syn) for 16 hours. The CM was spun down to remove cell debris and supplemented with apotransferrin (10 µg/ml), bovine insulin (10 µg/ml) and Sodium-selenite (220 nM) before adding it onto the oligodendroglial cell lines. Following the incubation, cells were processed for Western blotting or immunocytochemistry (ICC). The intracellular α-Syn signal (obtained by choosing the image plane beneth cell surface) was quantified using pro/image j software (Media Cybernetics Inc.) and represents fluorescence intensity across the entire cell area.

### Immunocytochemistry (ICC)

Cells were washed twice and fixed in paraformaldehyde (PFA) (4%). The detection of α-Syn within Oli-neu or OLN93 cells was performed using a human specific monoclonal anti α-Syn antibody, LB509 (1∶200 Invitrogen, Carlsbad, CA, USA) or Syn 303 (1∶3000, obtained from Virginia M.-Y. Lee, University of Pennsylvania, PA, USA). The secondary antibody was anti mouse-cy5 (Jackson laboratories, ME, USA). Slides were sealed with mounting medium (cat# M1289 Sigma Rehovot, Israel) and analyzed by confocal microscopy (see below).

### Western blotting

Detection of secreted α-Syn or β-Syn in CM. Naïve, α-Syn or β-Syn overexpressing MN9D cells were incubated to confluency in 100-mm dishes in standard growth medium. The medium was then replaced with fresh medium or serum-free medium supplemented with BSA and FA (at 50 and 250 µM, respectively) and cells were incubated for 16 h longer. The CM was collected and spun at 10, 000×*g* for 10 min. Aliquots were kept in a 4°C refrigerator and analyzed within 2–3 days.

*Detection of soluble α-Syn in MN9D cells* (including monomer and oligomers), wt or A53T mutant α-Syn over expressing cells were cultured as indicated and cells were then harvested and fractionated to collect the high speed cytosolic fraction (post 280,000×g) as previously described [33]. α-Syn oligomers detection was performed following incubation of protein samples at 65°C for 16–18 h for antigen retrieval [34].

Samples of collected CM (equal volume) or high speed supernatant of MN9D cells (equal protein amounts) were loaded on a 10% SDS-PAGE and following electrophoresis were transfered to PVDF membrane (Biorad Petach Tikva, Israel). The membrane was fixed with 0.4% PFA and blotted with either one of the following anti α-Syn antibodies: H3C (obtained from Julia George, University of Illinois, Urbana-Champaign, IL), LB509 (Invitrogen, Carlsbad, CA, USA) or SNL4 (obtained from Virginia M. Lee, see above). The blots were reacted with a secondary antibody-HRP conjugated and visualized with EZ-ECL system (Biological Industries, Beit Haemek, Israel), scanned with a Umax Magic Scan (Eastman Kodak, Rochester, NY, USA) and analyzed for density of α-Syn signal using UN-SCAN-IT GEL 3.1 software (Silk Scientific, Orem, UT, USA). The signal obtained for α-Syn in a specific sample of CM was normalized to the total amount of protein in the particular cultured dish.

*Detection of oligodendroglial gene expression in A53T α-Syn mouse brains*. One hemisphere of young (4–6 weeks old) mouse brain was homogenized in RIPA-buffer (Tris-HCl pH 7.6, 25 mM; NaCl 150 mM; NP-40 1%; Sodium deoxycholate 1%; and SDS 0.1%). The protein concentration was determined by the Bradford method [35]. Protein samples (10 µg) were separated by 10% SDS-PAGE, transferred to a PVDF membrane and probed with the following primary antibodies: CNPase (1∶1000, Sigma Rehovot Israel); PLP (1∶1000, Abcam Tel-Aviv Israel), CAII (1∶5000, kind gift from Said Ghandour, Louis Pasteur university, France), TPPP/P25 (1∶500, kind gift from Paul Henning Jensen, Aarhus University, Denmark) and visualized using the ECL detection system. The blots were scanned in a Umax Magic Scan (Eastman Kodak, Rochester, NY, USA) and analyzed for density of α-Syn signal *using UN-SCAN-IT GEL 3.1 software (Silk Scientific, Orem, UT, USA)*.

### RT PCR

Total RNA was isolated from one hemisphere of ntgt or A53T α-Syn mouse brains, naïve MN9D or Oli-neu cells using TRI Reagent (MRC, Cincinnati, OH, USA). The generation of cDNA was performed using High Capacity cDNA, Reverse Transcription Kit, (Applied Biosystems, Foster City, CA USA). Primer pairs were designed to exon-exon boundaries by Primer3 (v.0.4.0 software). For the detection of endogenous mouse α-Syn: 5′ primer sequnce 5-GTCTCAAAGCCTGTGCATCT-3 and 3′ primer sequence 5- TCCACACTTTCCGACTTCTG-3;

For NG-2 mRNA detection: 5′ primer sequence: 5-ACAGCTCCTGCCTCCTTCT- 3 and 3′ primer sequence 5- GCTGGGATGTGGAGAACTG -3;

For CNPase mRNA detection: 5′ primer sequence: 5- GCCCCGGAGACATAGTACC-3 and 3′ primer sequence: 5-GCGGGTAAAGCTTGTGTTATG-3;

For MBP mRNA detection: 5′ primer sequence: 5-CCAGCACCACTCTTGAACAC-3 and 3′ primer sequence: 5-CTCCATCCTTACTGGCCTTCT-3;

For PLP mRNA detection: 5′ primer sequence: 5-AGAACAGTGCCACTCCAAAGA-3 and 3′ primer sequence: 5-CAAAGACATGGGCTTGTTAG-3;

For TPPP/P25 mRNA detection: 5′ primer sequence: 5′-CGA GGA GGC TGT ACG TGA G-3′ and 3′ primer sequence 5′-GAG GAC ACA GCT TTC GTG AC-3′;

For CAII mRNA detection: 5′ primer sequence CCTCTGCTGGAATGTGTGAC-3′ and 3′ primer sequence 5-CAGCGTACGGAAATGAGACA-3′

Results were normalized against the expression levels of 18S. Analyses were performed using Applied Biosystems Prism 7300 sequence detection system with CYBR Green Master Mix (Applied Biosystems Foster City, CA USA).

### Clathrin silencing

Two different shRNAs with a reported effect on clathrin heavy chain (mouse) expression were used [36]. Cells were transfected with either one of the two clathrin shRNAs or a scrambled RNA sequence. Seventy-two hours post DNA transfection, 15 µg/ml of purified human α-Syn protein were added to the growth medium of the cells and incubation lasted for additional 16–18 hours. Cells were then processed for ICC using anti clathrin monoclonal ab (1∶200, BD Biosciences) and anti α-Syn polyclonal ab 7071 (provided by Peter Lansbury at Harvard Medical School, Boston, MA, USA), followed by secondary abs, anti mouse Alexa 488 (1∶300, Invitrogene, USA) and anti rabbit Cy5 (1∶300, Jackson laboratories, ME, USA)

### Immunohistochemistry (IHC)

Brain sections (6 µm) were deparaffinized in xylene followed by graded alcohol in descending ethanol concentrations. For α-Syn staining, antigen retrieval was performed by incubating the slides in 100% formic acid for 5 minutes, followed by extensive washes. Sections were blocked in 10% goat serum in 0.1 M Tris-HCl pH 7.6. Slides were then reacted with anti α-Syn antibody, Syn303 (1∶3000, obtained from Virginia M.-Y. Lee, see above), CAII (1∶500, provided by Said Ghandour, see above), TPPP/P25 (1∶100, provided by Poul Henning Jensen, see above), NeuN (1∶200, clone A60, Millipore, MA USA), GFAP (1∶200 DAKO, Glostrup, Denmark) or synaptophysin (1∶200, DAKO, Glostrup, Denmark). Slides were then washed and reacted with a second antibody, anti mouse Cy2 (1∶300, Jackson laboratories, ME, USA) and anti rabbit Cy5 (1∶300, Jackson laboratories, ME, USA). The stained sections were observed by confocal microscopy (see below). Quantifications were blinded for treatments. To reduce experimental error, quantifications were performed in sets of three slides, representing each of the three diet groups and comparisons were made only between slides that were stained and handled in parallel. To sum up the different comparisons and compare between the different staining events, we present the data as percent of the control, standard diet, set at 100% for each staining event as previously described [24]. Image series analyzed with Image pro plus 6.3 program (Media Cybernetics, Bethesda, MD, USA).

*Quantifying cell populations*. The selection of objects was done automatically by color definition. For objects specification, we used the smoothing options. The selected objects above 200 pixels for oligodendrocytes, 1000 pixels for neurons and 400 pixels for astrocytes were counted.

### Confocal microscopy

Was conducted using a Zeiss LSM 710 Axio Observer.Z1 laser scanning. The system is equipped with an argon laser 488 excitation with 494–630 nm pass barrier filter, Diode 405-30 laser excitation with 410–483 nm pass barrier filter and HeNe 633 lasers, excitation with 638–759 nm pass barrier filter. Fluorescence was collected simultaneously with differential interference contrast (DIC) images using a transmitted light detector. The fluorescence was collected by employing a “Plan-Apochromat” 63x/1.40 Oil DIC M27 or “Plan-Neofluar” 40x/1.30 Oil DIC M27 (Zeiss). In each experiment, exciting laser, intensity, background levels, photo multiplier tube (PMT) gain, contrast and electronic zoom size were maintained at the same level. For each antibody, the background was subtracted (determined by a negative control consisting of a secondary antibody alone). The zoom of each picture was obtained by choosing the plane with greatest fluorescent signal.

### Statistical analysis

Correlations coefficient was calculated using Microsoft Excell. P values<0.01 were considered significant. To compare between the three diet groups, the non-parametric Kruskal-Wallis ANOVA test was applied with Bonfferoni correction for multiple comparisons. Pair-wise comparisons between the low-DHA and high DHA groups at each mouse genotype were carried out using the non-parametric Mann-Whitney test.

## Results

### Intracellular expression and soluble oligomer levels affect α-Syn secretion from stably-transfected neuronal cells

**Figure 1:**
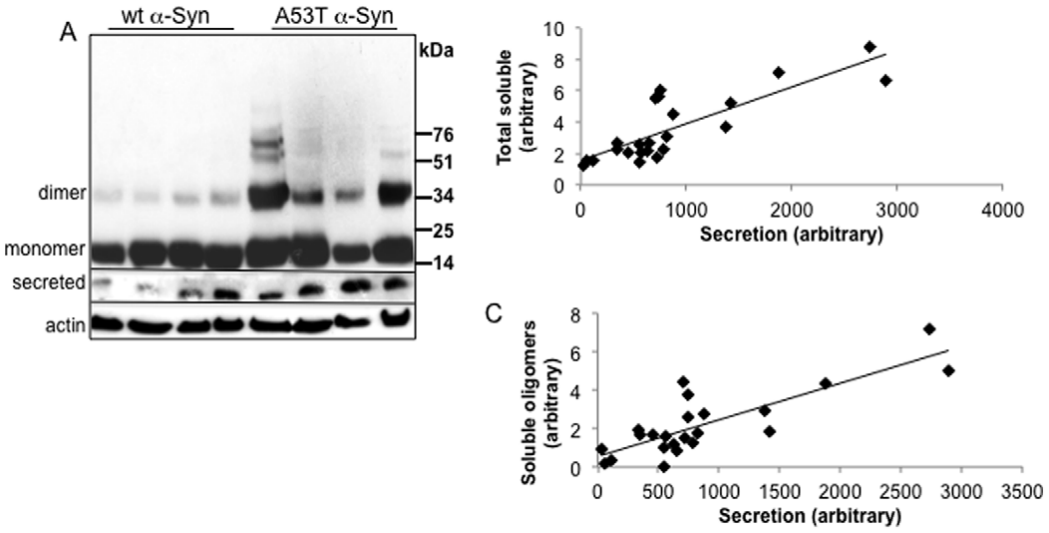
α-Syn secretion from MN9D neuronal cells positively correlates with its expression level and levels of soluble oligomers. A. Samples of the soluble fraction from different clones of MN9D cells stably expressing human wt or A53T α-Syn (15 µg protein) treated for oligomer detection at 65°C and the corresponding samples of collected conditioning medium (CM, 100 µl) were analyzed by Western blotting using the anti human α-Syn antibody, LB509. Equal protein loading was determined with anti actin antibody in samples of the soluble fraction. A representative blot. B. Graph representing the correlation of total soluble α-Syn (represented by the entire immuno-reactivity throughout the lane) plotted against secreted α-Syn; C. correlation of degree of soluble α-Syn oligomers (represented by the ratio of α-Syn oligomers to monomer) plotted against secreted α-Syn.

MN9D neuronal cells, stably expressing human wt or A53T α-Syn (8 and 4 different clones, respectively), were maintained in parallel from stable transfection to analyses. Each clone was analyzed by Western blotting for soluble α-Syn in the post high-speed cytosolic fraction (see methods) and in parallel, for secreted α-Syn in its conditioned medium (CM). The signal obtained on a Western blot for secreted α-Syn was quantified, normalized to the amount of cells in the particular dish (represented by protein concentration) and plotted against the signal obtained for total soluble α-Syn signal (the sum of signal obtained, including monomer and oligomers); or the degree of soluble α-Syn oligomers (the ratio of oligomers to monomer) and to actin levels. As expected, a higher degree of soluble α-Syn oligomers was detected for the A53T than wt α-Syn expressing clones (Fig. 1A). Plotting either total soluble α-Syn or degree of soluble α-Syn oligomers against secreted α-Syn resulted in a positive correlation, with a correlation coefficient r = 0.83, p<0.01 and r = 0.81, p<0.01 for total α-Syn and degree of α-Syn oligomers, respectively (Fig. 1B and C). Importantly, such positive correlations were also calculated for the eight different wt α-Syn clones, including high and low wt α-Syn expression levels (r = 0.77, p<0.01 and r = 0.78, p<0.01, for total α-Syn and degree of α-Syn oligomers, respectively). We concluded that both α-Syn expression levels and oligomer levels, within the soluble fraction, affect its secretion from cultured neuronal cells. That is, higher intracellular α-Syn expression and higher degree of oligomerization enhance secretion.

**Figure 2:**
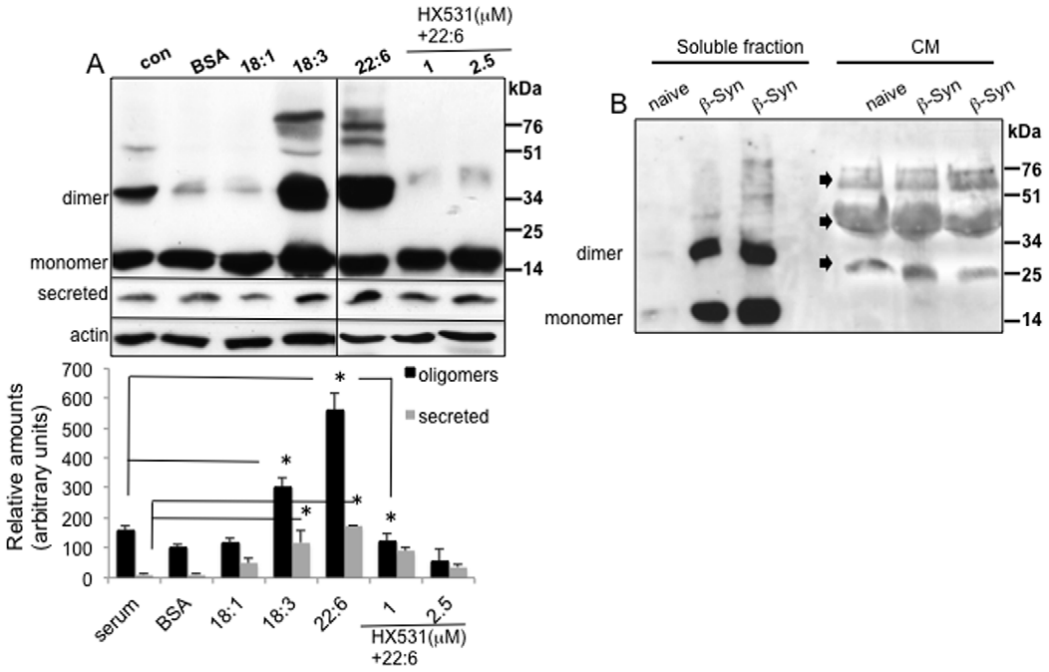
α-Syn secretion from MN9D dopaminergic cells is associated with PUFA-enhanced α-Syn oligomerization. A. Western blot analysis (as in Fig. 1A) of wt α-Syn over expressing MN9D cells. Sister cultures were treated in parallel either with serum-free DMEM supplemented with BSA only (at 50 µM) or together with the indicated fatty acids (at 250 µM), and the indicated concentrations of HX531, a specific antagonist for retinoic acid receptor (RXR) for 16 hours. Control cells were conditioned in standard conditioning medium. Bar graph represents quantification of signal obtained by Western blot analysis, mean ± SD of 2–4 repeats. 18∶1- oleic acid; 18∶3 α-linolenic acid; 22∶6, docosahexaenoic acid. B. Western blot analysis (as in 1A) of naïve and two different β-Syn over expressing MN9D clones, conditioned in standard serum-supplemented medium. The blot was reacted with an anti β-Syn antibody (BD-Transduction Laboratories). Arrows indicate non-specific bands.

As an alternative approach to alter the degree of α-Syn oligomerization, we utilized the enhancing effects of PUFAs on intracellular α-Syn oligomerization [24], [30], [33], [34], [37]. MN9D cells stably expressing wt α-Syn were cultured in a serum-free medium, supplemented with BSA only (50 µM) or with BSA (50 µM) bound to DHA (250 µM). Sixteen hours later, the CM and high-speed cytosolic fractions of respective cultures were collected and analyzed for secreted α-Syn and degree of α-Syn oligomers as above. Consistent with our previous observations, α-Syn oligomerization was not affected by 18∶1 MUFA, however, alpha linolenic acid (ALA, 18∶3) or DHA (22∶6) PUFAs enhanced α-Syn oligomers by ∼3 and 6 fold, respectively (Fig. 2). In accord with PUFA-enhanced α-Syn oligomerization, we detected higher levels of secreted α-Syn in the CM of PUFA-treated cells (Fig. 2). Specifically, α-Syn secretion from stably-transfected MN9D cells, treated with 18∶3 or 22∶6 PUFAs, was enhanced by 2–5 folds. Although there were high levels of variability between the different experiments, these differences in secretion rates were significant (ANOVA p = 0.023).

The enhancing effect of PUFA on α-Syn release could potentially result from two different effects. Namely, enhanced α-Syn oligomerization and pathology [24], [30], [33], [34], [37] or enhanced membrane trafficking [36], including exocytosis of α-Syn. To distinguish between the two potential mechanisms, we utilized a pharmacological compound, HX531, acting as a specific antagonist for retinoic X receptor (RXR) [38]. Recently, we have successfully shown that HX531 eliminated the effect of DHA on α-Syn oligomerization through its effect on RXR activation [24]. Yet, HX531 antagonist is not expected to affect membrane enrichment with PUFAs. Therefore, HX531 is used here to differentiate between DHA effect on α-Syn oligomers and its effect on membrane trafficking.

HX531 (at 1 and 2.5 µM) was added to α-Syn over expressing MN9D cells, cultured in medium supplemented with BSA alone or BSA with DHA (as above) for 16 hours. In accord with our previous observation, HX531 antagonist inhibited the enhancing effect of DHA on intracellular α-Syn oligomerization, resulting in oligomer levels similar to that found in BSA-treated, control cells (Fig. 2a). In accord, lower levels of secreted α-Syn were detected in sister cultures treated with HX531. Yet while oligomer levels were reduced to their levels in the control BSA- (or serum-) treated cells, levels of secreted α-Syn were still higher than control cells. This result indicated to us that PUFA-induced α-Syn secretion is promoted by both mechanisms, e.g. enhanced intracellular α-Syn oligomers levels and membrane trafficking.

To find out whether α-Syn secretion from cells that over express it is part of a specific mechanism or a result of cell death, we compared cell viability and secretion between different clones of α-Syn or β-Syn over expressing MN9D cells. The expression levels of the transfected proteins were determined by Western blotting and comparisons were made between 2–3 different clones with closely similar expression levels. We found no evidence for β-Syn secretion (Fig. 2b). Specifically, while the occurrence of α-Syn was clearly detected in the CM (see above) no signal for β-Syn was obtained in the CM. The result was obtained with a specific anti β-Syn antibody (BD-Transduction Laboratories NJ, USA) and verified with H3C antibody, recognizing both synuclein homolog (not shown). Importantly, we did not detect significant differences in cell viability between the α-Syn- and β-Syn-over expressing MN9D cells. We therefore concluded that the cells secrete α-Syn protein as part of a specific mechanism.

### Oligodendroglial cell lines take up α-Syn from their growth medium

**Figure 3:**
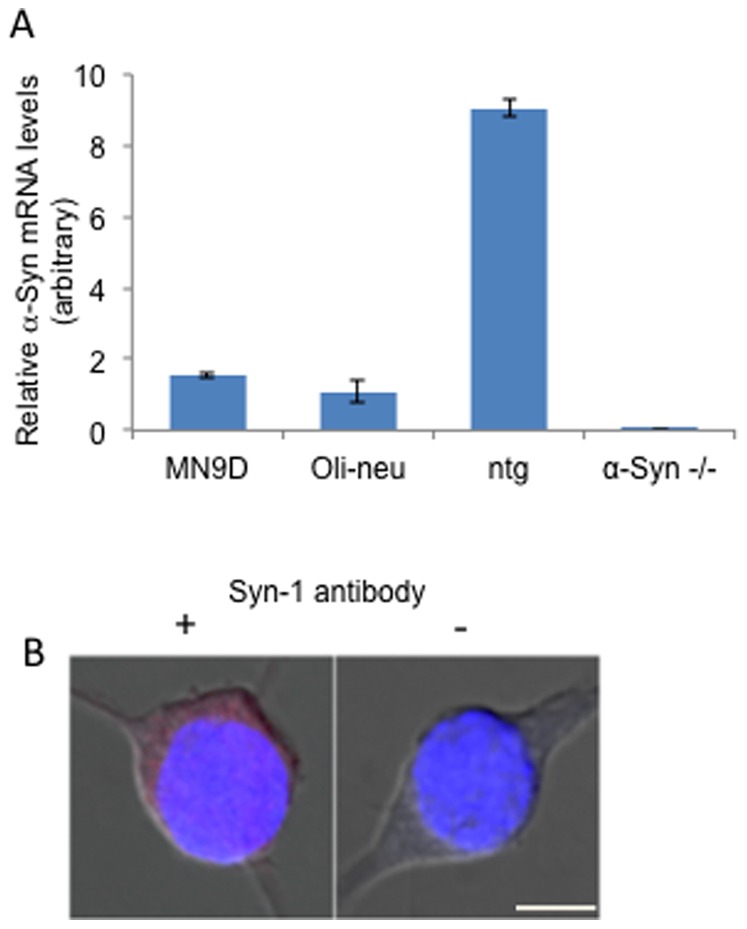
α-Syn is normally expressed in Oli-neu cells. A. Real time PCR (rtPCR) using specific primers designed to detect endogenous α-Syn in naïve MN9D and Oli-neu cells, non-transgenic (ntg) and α-Syn −/− whole mouse brain. B. ICC of cultured naïve Oli-neu cells using anti α-Syn antibody, Syn-1. Scale bar = 10 µm.

To investigate whether oligodendroglial cells take up α-Syn from their growth medium, two cell lines with oligodendroglial properties were used, namely, Oli-neu cells [31] and OLN-93 cells [32]. We first determined endogenous α-Syn expression by rtPCR and ICC. A specific signal for α-Syn mRNA was detected by rtPCR in Oli-neu cells. The α-Syn mRNA level detected in Oli-neu cells is similar to the level detected in MN9D dopaminergic cells, while whole ntg mouse brain has an approximately ten-fold higher expression level (Fig. 3A). No signal for α-Syn mRNA was detected in brain tissue of α-Syn −/− mice [26]. In accord with the presence of α-Syn mRNA, a low and antibody-specific signal for endogenous α-Syn was obtained in Oli-neu cells by ICC using the anti syn-1 antibody (Fig. 3B). Similar results were obtained with H3C antibody (not shown).

To determine whether Oli-neu cells take-up α-Syn from their environment, we incubated naïve Oli-neu cells in CM collected from MN9D dopaminergic cells overexpressing human α-Syn or supplemented the growth medium of Oli-neu cells with purified recombinant human α-Syn.

**Figure 4:**
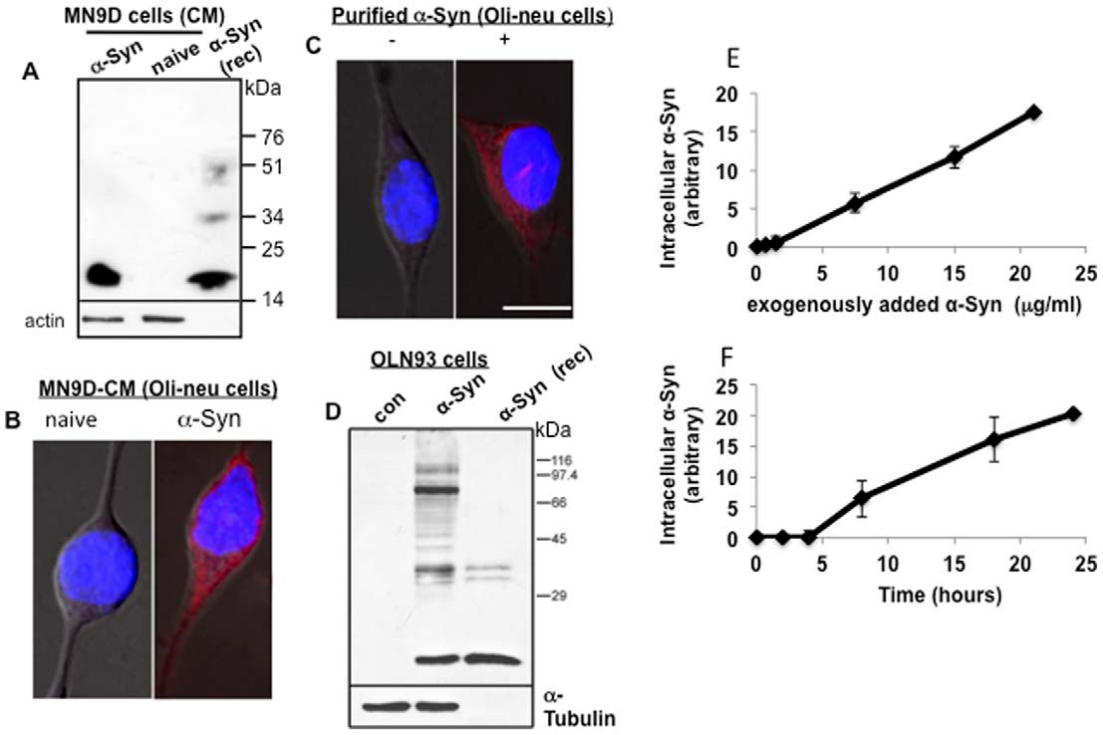
Oli-neu and OLN-93 oligodendroglial cells take up α-Syn from their environment. A. Samples of conditioned medium (CM) collected from naïve and α-Syn over expressing MN9D cells, analyzed by Western blotting using LB509 antibody. B. ICC of Oli-neu cells incubated for 18 hours in CM collected from naïve or α-Syn over expressing MN9D cells (demonstrated in A) using LB509 ab. C. ICC as in (B) following 6 hours incubation without or with 15 µg/ml purified human α-Syn in the growth medium. D. Western blot analysis of OLN93 cells cultured for 6 hours in standard growth medium, supplemented without (con) or with 10 µg/ml purified α-Syn. The blot was reacted with SNL-4 antibody. E. Uptake of purified α-Syn by Oli-neu cells is dose dependent. Cells were incubated with 0–21 µg/ml purified α-Syn in their growth medium for 16 hours and then processed for α-Syn detection by ICC using anti α-Syn antibody, LB509 (Mean ±SD of n = 20 cells). F. Uptake of purified α-Syn by Oli-neu cells is time dependent. Oli-neu cells incubated with 15 µg/ml purified α-Syn in their medium for 0–24 hours and processed for intracellular α-Syn detection as in (E).

The amounts of secreted α-Syn in CM collected from MN9D cells, naïve and overexpressing α-Syn, were determined by Western blotting. Importantly, although low α-Syn mRNA levels were detected in naïve MN9D cells (Fig. 3A), we could not detect secreted endogenous α-Syn protein in CM collected from these cells (Fig. 4A). Oli-neu cells were incubated for sixteen hours in CM collected from naïve and α-Syn overexpressing MN9D cells, as specified. Cells were then processed for intracellular α-Syn detection by ICC, using the anti human α-Syn antibody, LB509. Results were verified with an additional anti human α-Syn antibody, generated in our lab. The presence of a specific immunoreactive signal for α-Syn, obtained with the anti human α-Syn antibodies, was clearly demonstrated. The signal was present in cytoplasm and nuclei of Oli-neu cells cultured in CM collected from α-Syn over expressing MN9D cells. No signal for human α-Syn was detected in Oli-neu cells cultured in parallel, in CM collected from naïve MN9D cells (Fig. 4B).

Next we tested uptake of exogenously added human α-Syn by cultured Oli-neu. Cells were cultured in standard growth medium, supplemented with purified human α-Syn (15 µg/ml) for 16 hours and then processed for the detection of α-Syn by ICC. A specific α-Syn immunoreactive signal was detected in Oli-neu cells cultured with the purified human α-Syn, which was not seen in sister cultures grown incubated in standard growth medium without α-Syn (Fig. 4C). Similar results, representing uptake of exogenously added α-Syn, were obtained with OLN-93 cells (Fig. 4D). Specifically, OLN93 cells were cultured with purified human α-Syn (10 µg/ml) for 6 hours and then processed for detection of α-Syn by Western blotting, using anti α-Syn SNL4 antibody. α-Syn monomer and oligomers were readily detected in those cells incubated in the presence of purified α-Syn and not in control cells conditioned in parallel, without purified α-Syn.

Uptake of purified human α-Syn by Oli-neu cells was measured following 16 hours of incubation with 0–21 µg/ml purified α-Syn; or incubation with 15 µg/ml purified α-Syn for 0–24 hours. Following these incubations, cells were processed for ICC with the anti human α-Syn antibody, LB509. Quantifying the specific α-Syn signal obtained within Oli-neu cells indicated a time and a dose dependent uptake of the exogenously added α-Syn protein (Fig. 4E, and F). Specifically, a linear graph representing α-Syn uptake was obtained by incubating the cells for 16 hour with 0.7–21 µg/ml (R^2^ = 0.945), representing a dose-dependent uptake. Similarly, a linear graph, representing α-Syn uptake was obtained for Oli-neu cells incubated for 4–24 hours in the presence of 15 µg/ml α-Syn (R^2^ = 0.931), representing a time dependent uptake.

### Uptake of α-Syn by primary cultures of rat brain oligodendrocytes

**Figure 5:**
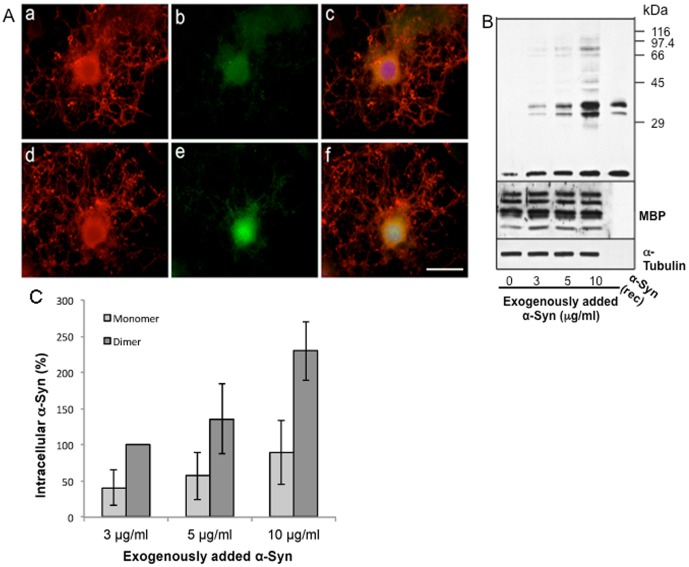
Primary rat oligodendrocytes take-up purified α-Syn from their growth medium in a concentration-dependent manner. A. Oligodendrocytes (6 div) prepared from rat brains were incubated either without (a–c) or with 10 µg/ml purified α-Syn for 6–7 h (d–f) and processed for immunocytochemistry using antibodies against MBP (a,d; red) or α-Syn (SNL-4, b,e; green). Overlay with DAPI (c,f; blue). Bar, 20 µm. B. Rat brain oligodendrocytes were incubated with 3, 5, or 10 µg/ml purified α-Syn as indicated on the bottom, for 24 hours. Cell lysates (10 µg of protein) were analyzed by Western blotting using anti α-syn (SNL-4), myelin basic proteins (MBP) and α-tubulin antibodies, as indicated on the right. C. Quantitative evaluation of α-Syn mono- and oligomers was carried out by densitometric scanning of the blots. Results show the mean +/− SEM from three independent experiments. Monomeric α-Syn levels are given as percentage above the level of endogenous α-Syn, the oligomeric level after incubation with 3 µg/ml human recombinant α-Syn was set at 100%.

To confirm that oligodendrocytes are capable of taking-up α-Syn from their environment, primary cultures of oligodendrocytes (6 div) derived from rat brains were incubated with or without 10 µg/ml α-Syn for 6–7 hours. Indirect immunofluorescence and Western blot analysis revealed a detectable basal level of α-Syn expressionin primary oligodendrocytes using the anti α-Syn SNL4 antibody (Fig. 5A, B). α-Syn uptake by rat brain oligodendrocytes was observed in the cell body and nucleus (Fig. 5A). Western blot analysis and quantitative evaluation of the immunoblots by densitometric scanning further demonstrated that the uptake of α-Syn by primary oligodendrocytes is dose dependent between 3–10 µg/ml and that in the cell lysates it is detectable in its monomeric and oligomeric forms (Fig. 5B, C).

### α-Syn uptake by cultured oligodendroglia is clathrin-dependent

**Figure 6:**
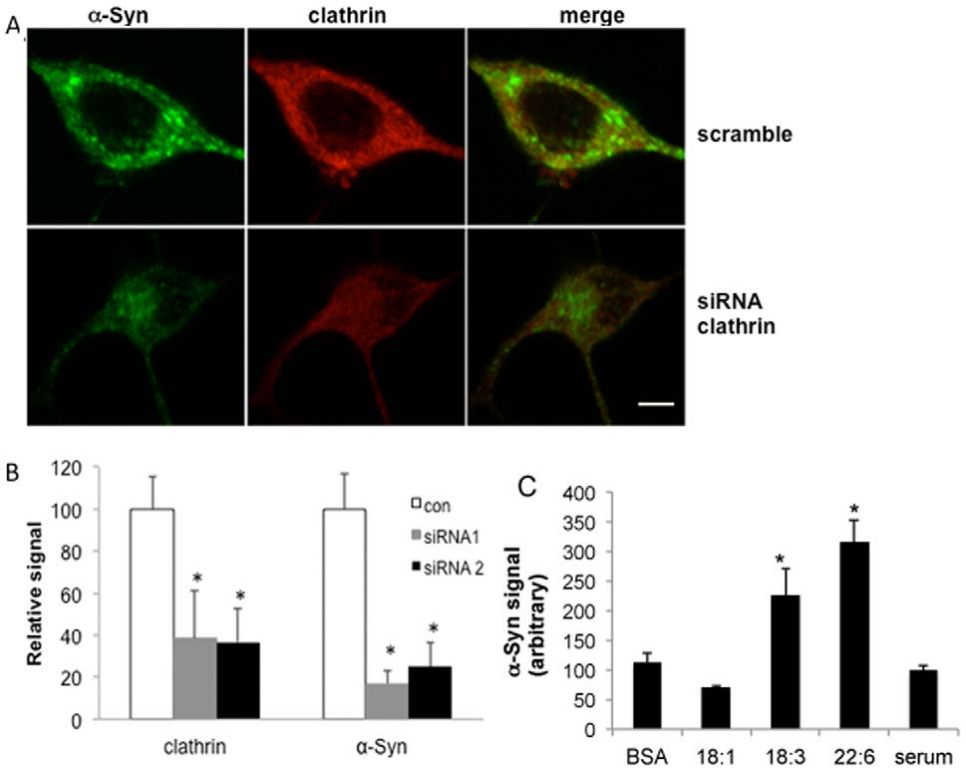
α-Syn uptake by Oli-neu cells is dependent on clathrin expression and enhanced by exposure to PUFAs. A. Naïve Oli-neu were transiently transfected either with scramble or two different shRNAs designed against clathrin heavy chain. 56 hours post DNA-transfection, cells were cultured in standard growth medium supplemented with purified human α-Syn (at 15 µg/ml) for 16 hours and processed for ICC with anti clathrin and anti α-Syn antibodies. Bar, 10 µm. B. Graph representing quantitation of the signal obtained by ICC, mean ± SD of n = 20–30 cells in each treatment. *, p<0.001 ANOVA. C. Naïve Oli-neu cells (sister cultures) were incubated in the presence of purified α-Syn (at 15 µg/ml) added to standard growth medium (with serum); serum-free DMEM supplemented with BSA only (at 50 µM); or BSA together with the indicated FA (at 250 µM) for 16 hours. Cells were then washed and processed for ICC for the detection of intracellular α-Syn using anti the α-Syn antibody, LB509. The signal within cells was quantified. Mean ±SD of n = 10–15 cells in each treatment. *, P = 0.0034 (ANOVA).

To assess the involvement of clathrin in α-Syn uptake by oligodendroglial cells, we transiently transfected naive Oli-neu cells with either one of two different shRNAs designed to silence the expression of clathrin heavy chain or a third construct containing a scrambled sequence. These shRNAs were shown to effectively silence clathrin expression [36]. 56 hours post transfection, purified α-Syn (15 µg/ml) was added to the growth medium of the transfected cells and incubation continued for an additional 16 hours. Cells were then processed for ICC to detect α-Syn and clathrin using specific antibodies (Fig. 6). Quantifying the signal obtained by ICC, we confirmed a significantly lower signal for clathrin in cells transfected with the clathrin shRNAs. That is, the measured signal for clathrin was 36.6±15.8% for shRNA1-transfected cells and 38.2±11.3% for shRNA2-transfected cells of the signal obtained for control cells, transfected with the scrambled sequence and set at 100% (mean ± SD of n = 20–30 cells). In accord with the lower clathrin expression levels, we measured a significantly lower α-Syn signal within Oli-neu cells, representing lower uptake. Specifically, considering α-Syn signal obtained in Oli-neu cells transfected with the scrambled sequence as 100%, the signal measured for α-Syn was 17.5±6.3% and 37.7±16.3% for shRNA 1 and shRNA 2, respectively (mean ± SD of n = 20–30 cells; P = 0.0002 ANOVA) (Fig. 6A and B).

### α-Syn uptake in Oli-neu cells is affected by exposure to polyunsaturated fatty acids (PUFAs)

In previous studies, we have shown that membrane PUFA composition and clathrin mediated endocytosis (CME) are affected by exposure of cultured cells to physiological concentrations of PUFAs [36], [39]. To find out whether PUFAs alter α-Syn internalization into Oli-neu cells, we incubated the cells in the presence of 15 µg/ml exogenously added, purified human α-Syn for 16 hr in a serum-free medium supplemented either with BSA alone (at 50 µM) or together with FAs (at 250 µM). Control cells were conditioned in standard serum-supplemented medium. The signal detected within Oli-neu cells was quantified by ICC with anti human α-Syn ab LB509 (Fig. 6C). Setting the signal obtained for control cells at 100%, we found that incubating Oli-neu cells in 18∶3 PUFA-supplemented DMEM, resulted in a higher degree of α-Syn internalization. Specifically, the measured α-Syn signal in Oli-neu cells treated with 18∶3 (227.9±42.5%) was significantly higher than the signal measured for BSA alone (112.1±15.9%) or with 18∶1 monounsaturated fatty acid (MUFA) (69.0±2.9%). This signal represents mean ± SD of n = 10–15 cells with P = 0.0034 (ANOVA). A stronger signal for α-Syn uptake was determined in cells conditioned in the presence of DHA (22∶6, 316.6±37.5%) (P = 0.0022, ANOVA n = 20–30 cells). The signal measured for cells treated with BSA only or BSA-18∶1 was not significantly different than serum treated cells (Fig. 6C). We therefore concluded that membrane enrichment of Oli-neu cells with PUFAs enhances α-Syn uptake and internalization.

### Evidences for affected oligodendrocytes in brains of A53T α-Syn tg mice modeling neuronal synucleinopathies

To in vivo test the hypothesis that neuronal secreted α-Syn underlies its pathogenic accumulation in oligodendrocytes in MSA, we first determined whether the A53T α-Syn tg mouse line [25] depict oligodendroglial pathology, and thus may serve as a model for MSA. These mice express α-Syn under the PrP promoter and originally were described as a model for neuronal synucleinopathies. While prion protein is mostly expressed in neurons, a portion of it is also expressed in glia cells, including oligodendroglia [40].

**Figure 7:**
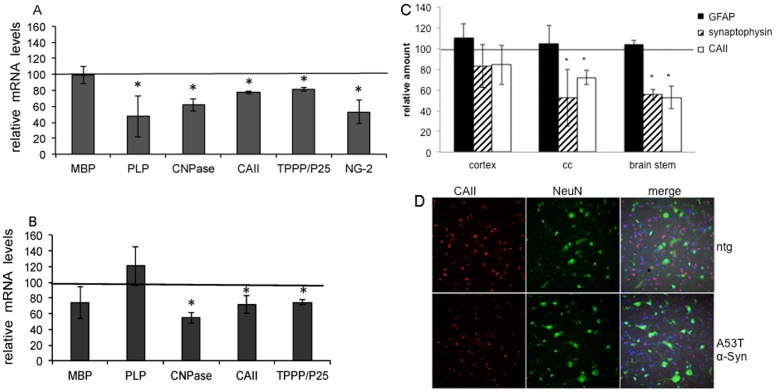
Oligodendrocyte are affected in A53T α-Syn tg mouse brains. **A.** Total RNA was extracted from one mouse brain hemisphere (n = 4–5 brains) of ntg and A53T α-Syn and analyzed by real time-PCR with specific primers as indicated in methods. The results are presented as a percent of the 100% signal detected for each gene in the ntg mouse brains (represented by the vertical bar). MBP, myelin basic protein; PLP, proteolipid protein; CNPase, 2′, 3′-Cyclic Nucleotide 3′-Phosphodiesterase; CAII, carbonic anhydrase II; TPPP/P25, tubulin polymerization promoting protein; and NG-2 chondroitin sulfate proteoglycan. *, P<0.01, ANOVA. **B.** Samples of protein extracts from one brain hemisphere (n = 4–5) of ntg and A53T α-Syn mice, analyzed by Western blotting using specific antibodies to the indicated proteins (as in A). Blots were quantified using UnScan it gel 6.1. The results are presented as a percent of the 100% signal detected for each protein in ntg mouse brains (represented by the vertical bar). *, p<0.01, ANOVA. C. Graph representing quantifications of GFAP, synaptophysin and CAII signals by IHC in paraffin sections from ntg (100%, bar) and A53T α-Syn mouse brains. N = 4–5 mouse brains with 3–5 fields for each mouse brain, mean ± SD, *, P<0.01, ANOVA. D. A representative image of gigantocellular nuclei in brain stem of ntg and A53T α-Syn tg mouse brain obtained by IHC. Paraffin sections stained with TPPP/P25 (oligodendrocytes, red) and NeuN (neurons, green) and counter stained with DAPI (blue). Bar, 50 µm.

To this aim, the expression levels of specific oligodendrocyte-encoded genes were analyzed in A53T α-Syn in comparison to ntg mice (C57Bl). Total RNA was extracted from one mouse brain hemisphere (n = 4–5 brains) and the mRNA levels of the following oligodendrocyte-specific genes were evaluated by rtPCR in relation to 18S RNA levels: myelin basic protein (MBP) and proteolipid protein (PLP) as major structural components of the myelin sheaths; 2′, 3′-Cyclic Nucleotide 3′-Phosphodiesterase (CNPase), a protein enriched in myelin and oligodendrocytes; carbonic anhydrase II (CAII) expressed in the cytosols of oligodendroglia; tubulin polymerization promoting protein (TPPP/P25), an oligodendroglia specific protein with its expression being initiated at the time of myelination; and chondroitin sulfate proteoglycan (NG-2), a marker for oligodendroglia precursor cells. Setting the levels detected for ntg mice at 100%, we measured lower levels of expression for PLP (47%, p = 1.53E-06), CNPase (61%, p = 1.45E-06), CAII (77%, p = 0.002), TPPP/P25 (81%, p = 6.46E-05) and NG-2 (52%, p = 1.07E-05) in young A53T α-Syn mouse brains, Yet no differences were detected in mRNA levels of MBP (Fig. 7A).

To compare expression at the protein level we utilized the second mouse brain hemisphere to extract protein (see methods) and quantified their amounts by Western blotting using specific antibodies. Comparing the protein levels, we detected ∼54–74% significantly lower levels for CNPase, CAII and TPPP/P25 in A53T α-Syn brains than the ntg controls. No differences in the levels of the myelin proteins, PLP and MBP, were detected in the A53T mouse brains (n = 4–6 mouse brains for each genotype, p<0.01, ANOVA) (Fig. 7B).

To determine whether the lower expression levels of specific oligodendrocyte genes (described above) are accompanied by a loss of oligodendrocytes, we quantified oligodendroglia, astroglia and neurons in paraffin sections of young ntg and A53T α-Syn mouse brains (n = 4 for each genotype). Specifically, we stained in parallel, for oligodendroglia (CAII or TPPP/P25), astroglia (GFAP), and neurons (NeuN). The relative amounts of the different cell populations were quantified in three different brain regions, cortex, corpus callosum and gigantocellular nuclei in brain stem. Consistent with the original description of this mouse model, no difference in the number of neurons, determined by NeuN staining, was detected between ntg and A53T α-Syn mice in the three specific brain regions tested [25]. In addition, no evidence for astrocytic gliosis was found in these three brain regions. In contrast, lower signals for either one of the oligodendrocytes protein markers tested, CAII or TPPP/P25, were detected in A53T α-Syn than ntg mouse brains. Specifically, the number of oligodendrocytes per slide stained with CAII was 102±7.2 and 72±11.2 in the corpus callosum of ntg and A53T α-Syn mice, respectively, and 65.5±8 and 44.3±8 in the brain stem of ntg and A53T α-Syn mice, respectively (n = 4–5 mice, p<0.01, ANOVA) (Fig. 7C). The differences in number of oligodendrocytes in the cortex did not reach significancy. To verify the results obtained with CAII, we stained adjacent sections with the same antibody set up but using TPPP/P25 as a marker for oligodendrocytes and obtained highly similar results (Fig. 7D).

In accord with the lower number of oligodendrocytes in brain stem and corpus callosum of young A53T α-Syn mice, we detected evidence of synaptic injury, represented by a lower signal for synaptophysin in these brain regions (Fig. 7C). Specifically, the measured signal for synaptophysin in brain stem and corpus callosum of A53T mice was 45.4±15.7% and 45±3% (p<0.01 ANOVA) respectively, of the signal obtained for ntg mice (set at 100%). These results indicate that the A53T α-Syn mice, originally described as a model for neuronal synucleinopathies such as PD, also harbor insults characteristic of MSA, with affected oligodendrocytes and synaptic injury.

### Dietary brain DHA levels affect neuronal and oligodendroglial α-Syn pathology in A53T α-Syn mouse brains

In a recent study, we have shown that dietary alterations in brain DHA levels affect the degree of α-Syn -related pathology in brains of A53T α-Syn mice [24]. We now extended this study to investigate the effect of brain DHA levels on α-Syn localization to oligodendrocytes. For this aim, we utilized our recent protocol for dietary manipulations in brain DHA composition [24]. Specifically, A53T α-Syn mice were fed either one of the following diets, a low-DHA (and low n-3 PUFA); a high-DHA (0.67% DHA); or a standard mouse chow. The diets were administrated for 230 days and mice were sacrificed at 9–10 months of age. Comparisons for the effects of DHA were performed between the high and low DHA diets after normalization to the standard chow.

**Figure 8:**
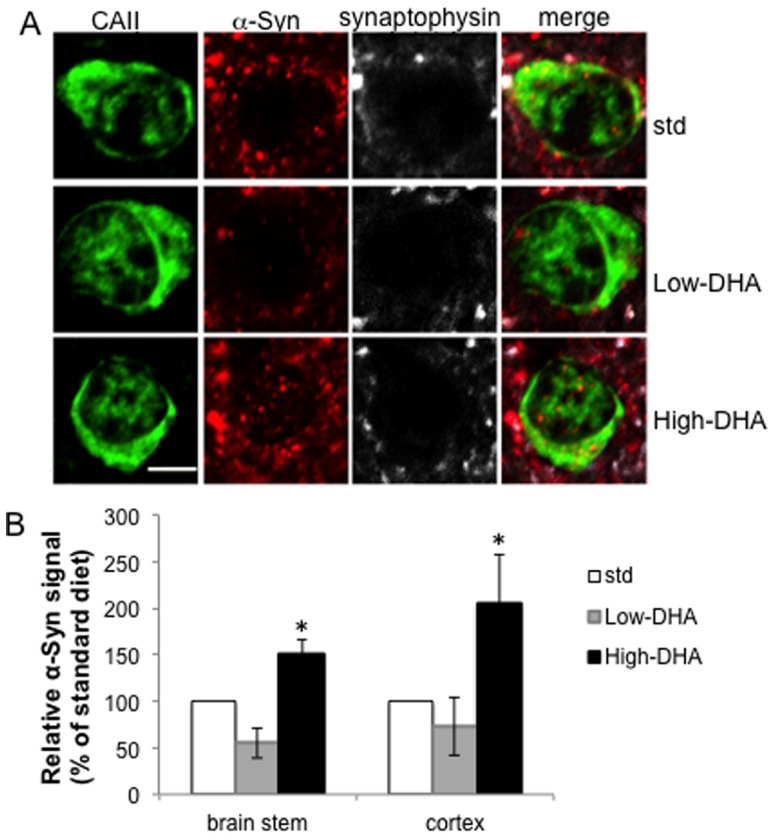
Dietary alterations in brain DHA levels affect α-Syn accumulation in oligodendrocytes in brains of A53T α-Syn mice modeling MSA. A53T α-Syn mice were fed for 230 days either one of the three diets, a low-DHA (and low n-3 PUFA); a high-DHA (0.67% DHA); or a standard mouse chow. A. IHC of paraffin sections showing the Gigantocellular nuclei in brain stem, stained in parallel for CAII (oligodendrocytes, green); α-Syn (red) and synaptophysin (white). B. Graph representing the α-Syn signal in co-localization with TPPP/P25 but not with the synaptophysin signal. Relative results (normalized to the standard chow group, designated as 100%) for the low and high DHA diets. Images were quantified with the Image pro plus 6.3 program (Media Cybernetics, MD, USA). Mean ± SD of n = 6; *, p<0.001 ANOVA. Bar, 10 µm.

Following upon our recent findings, we now attempted to determine the potential effect of alterations in levels of neuronal α-Syn pathology on its localization to oligodendrocytes by IHC, measuring α-Syn signal co-localizing with specific protein markers in paraffin brain sections. For this aim, we triple stained coronal brain sections with specific antibodies against α-Syn, CAII and synaptophysin. To verify the results, we repeated stainings with a similar setup but with TPPP/P25 antibody for oligodendrocytes. We focused on two brain regions, brain stem and cortex, containing high and low levels of α-Syn cytopathology, respectively [24], [25]. The results indicated a higher degree of α-Syn immunoreactivity detected within oligodendrocytes of A53T α-Syn mice fed the high DHA diet and lower α-Syn immunoreactivity with the low DHA diet. A significant difference in α-Syn signal detected by IHC and co-localized with the oligodendroglial markers, CAII, was obtained by comparing brain stems of mice from the low and high DHA diet groups (Fig. 8). Specifically, the relative α-Syn signal in oligodendrocytes measured and normalized to the standard chow group (designated as 100%) for the low and the high DHA diets were 55.5±32.3% and 151±30.2% respectively (mean ± SE; n = 6 mice per group; p<0.01, ANOVA) (Fig. 8). Similar results were obtained in the cortex of these mice. Specifically, the relative α-Syn signal measured and normalized to the standard chow group (designated as 100%) for the low and the high DHA diets were 45±8.7% and 274±12.0%, respectively, (mean ± SE; n = 4–6 mice per group; p<0.01, ANOVA). The effects of DHA-enhanced α-Syn neuropathology on α-Syn localization to oligodendrocytes were verified with a second antibody, TPPP/P25, with highly similar results (8B).

To exclude the possibility that dietary DHA affects α-Syn expression in oligodendrocytes through enhanced PrP promoter transcription activity, we measured the mRNA levels of transgenic human α-Syn and endogenous PrP by real-time (RT) PCR. Specifically, total RNA was extracted from one hemisphere of A53T α-Syn mice, from each of the three tested diet groups (n = 4 mice for each group). We found no evidence for enhanced PrP promoter transcription activity in brains of mice fed the high vs. low DHA diets. Likewise, no evidence for an effect of the diet on mouse endogenous α-Syn expression was found [24]. Moreover, treating N2a cells with DHA at 50–200 µM for 16–24 hours had no enhancing effect on PrP mRNA levels (not shown). We therefore concluded that the effect of dietary DHA on α-Syn localization to oligodendrocytes results from enhanced neuronal α-Syn pathology and is not a result of enhanced transcription mediated by the PrP promoter.

## Discussion

The accumulation of α-Syn protein in oligodendrocytes as a mechanism underlying neurodegeneration in the synucleinopathy, multiple system atrophy (MSA) was investigated. The results obtained in cultured neuronal MN9D cells, over expressing α-Syn, indicate that intracellular α-Syn protein level and the degree of soluble α-Syn oligomers affect its secretion. Our data demonstrate that oligodendroglial cells are capable of taking up α-Syn from their environment, either from medium conditioned by neuronal cells or from their growth medium supplemented with recombinant human α-Syn. Moreover, the uptake by oligodendrocytes is dependent on clathrin expression.

To investigate the effect of intra-neuronal α-Syn pathology on its accumulation in oligodendrocytes *in vivo*, we measured α-Syn localization in oligodendrocytes following dietary alterations in brain docosahexaenoic acid (DHA) levels, in mice transgenic for the A53T α-Syn mutant form. In accord with our recent report describing enhanced levels of α-Syn-related pathology following high DHA diet and the reverse effect with a low-DHA diet, we now report higher amounts of α-Syn immunoreactivity within oligodendrocytes in brain sections of mice fed the high-DHA than those fed the low-DHA diets. Hence, enhanced neuronal α-Syn pathology may be causally related to its accumulation in oligodendrocytes in MSA.

The A53T α-Syn tg mouse line was originally described as a model for synucleinopathies [25]. These mice express human A53T α-Syn under the control of the PrP promoter. The mice develop an age-dependent disease, characterized by motor impairment leading to paralysis and death. In accord with phenotype appearance, the mice develop intra-cytoplasmic, neuronal, α-Syn–positive inclusions. The α-Syn- pathology is abundant in the brain stem and in several nuclei affected in PD and DLB, such as the locus coeruleus and the raphe nucleus, and also in the striatum. Yet, no α-Syn-pathology was originally described in oligodendrocytes in this mouse model. In addition, no evidence for neuronal loss, including for tyrosine hydroxylase-positive neurons, was originally reported for this A53T α-Syn tg mouse line. We detected evidences for synaptic injury, represented by lower levels of synaptophysin immunoreactivity in the two brain regions tested, the corpus callosum and brain stem. The lower expression levels of oligodendrocyte-specific genes and the lower density of oligodendrocytes we detected in brains of young A53T α-Syn tg mice suggest that oligodendrocytes are affected in this mouse model. Several mouse models for MSA were recently generated, specifically directing the expression of α-Syn to oligodendrocytes using specific promoters of oligodendroglial genes (see for example [41], [42], [43], [44]). These models were shown to recapitulate features of MSA and are highly useful for elucidating pathways of α-Syn toxicity in oligodendrocytes, yet, these models can not explain the mechanisms involved in the pathogenic accumulation of α-Syn within oligodendrocytes.

It is currently unclear whether α-Syn secretion is part of a pathogenic process or rather relates to the physiological function of this protein. This may be represented by the difficulties to distinguish healthy individuals and PD patients based on their CSF levels of α-Syn [45], [46], [47], [48], [49], [50], [51], [52], [53]. In this regard, the secretion of α-Syn appears to be a specific mechanism and does not occur for β-Syn homolog. We report a positive correlation between the levels of intraneuronal α-Syn oligomers and its secretion. Higher oligomer levels and higher amount of secreted α-Syn are detectable in cultured cells stably expressing the A53T mutant form than wt α-Syn expressing cells. Furthermore, we found a positive correlation between the total amount of intracellular α-Syn and the amount of secreted α-Syn, detected in the CM. α-Syn dosage is strongly associated with the disease. Duplications and triplications in α-Syn gene locus, translating to elevated levels of α-Syn expression were shown to cause familial PD [54]. It is therefore interesting to find out whether α-Syn secretion from neuronal cells is enhanced in these patients.

The mechanism of α-Syn secretion was shown to involve vesicle trafficking that is independent of endoplasmic-reticulum (ER)-Golgi apparatus. α-Syn was detected within the lumen of vesicles [7] and is released by exosome-like vesicles in a Ca^2+^ dependent manner [8]. Secreted α-Syn was shown to affect the recipient cells. Specifically, uptake of neuronal-secreted α-Syn was shown to affect the viability of neighboring neuronal cells and alters gene expression patterns in astrocytes, causing an inflammatory response [8], [9]. The significance of α-Syn release and its uptake by neighboring cells is not fully understood, yet it may underlie the mechanism of pathogenic α-Syn spread as suggested by the Braak hypothesis [55]. Recently, it was suggested that α-Syn pathology may propagate throughout the brain in a prion like mechanism [13]. In this study we suggest that the pathogenic spread of α-Syn may also underlie mechanisms leading to MSA.

In this study we focus on the effect of enhanced accumulation of neuronal α-Syn on its secretion and subsequent uptake by oligodendroglial cells. However, it is plausible that additional cell populations, including astrocytes, microglia and certain neuronal cells, are similarly affected.

We utilized the effect of dietary brain DHA levels as a mean to control the degree of α-Syn pathology in-vivo, in brains of A53T α-Syn tg mice. Recently, we have demonstrated that α-Syn related pathology positively correlate with brain DHA contents. The accumulation of soluble and insoluble, neuronal α-Syn, astrocytic gliosis and synaptic injury, were affected by these dietary DHA alterations [24]. The results herein may represent an extension of the previous study, indicating that dietary brain DHA levels control an additional aspect of α-Syn pathology, namely, its accumulation in oligodendrocytes in MSA. Together, our previous and current results suggest that α-Syn associations with PUFAs and specifically, its associations with DHA, are involved in a spectrum of mechanisms implicated in α-Syn-pathophysiology.

Two apparently different mechanisms may underlie the effect of DHA and additional PUFAs on α-Syn secretion: 1) their previously shown effect to enhance the levels of intraneuronal cytotoxic α-Syn forms through activation of nuclear receptors [24]; 2) their enhancing effect on membrane trafficking, including endocytosis and exocytosis and enhanced synaptic vesicle recycling [36]. To determine which of the two mechanisms is involved in α-Syn secretion we utilized the specific RXR antagonist, HX531. This antagonist enables to eliminate DHA-enhancing effect on α-Syn oligomerization [24]. Thus, α-Syn secretion from cells treated with DHA together with HX531 is potentially affected by DHA-dependent membrane trafficking only. The results indicate that α-Syn secretion is affected by both mechanisms, intracellular α-Syn oligomers levels and membrane trafficking. α-Syn secretion from cultured cells treated with DHA together withHX531 are lower than from DHA without HX531, yet higher than BSA treated-control cells.

We show that α-Syn uptake by cultured oligodendrocytes is inhibited by silencing the expression of clathrin heavy chain, indicating the potential involvement of clathrin mediated endocytosis in this process. The involvement of endocytosis-dependent uptake of α-Syn was previously shown in neuronal and glial cells suggesting specific rather than non-specific uptake. Yet, the mechanism of endocytosis varies between cell types. α-Syn internalization, affected by a dynamin dominant negative, was previously suggested for the uptake of fibrillar and aggregated but not monomeric α-Syn in neuronal cells [12]. The involvement of dynamin in α-Syn uptake was shown also in astroglial cells [9]. In microglia, monomeric α-Syn was shown to internalized via GM1-mediated endocytosis and required intact lipid rafts. The uptake of α-Syn into microglia was independent of clathrin-, caveolae-, or dynamin-dependent endocytosis [56]. Others have shown that oligomeric α-Syn internalizes into microglia more efficiently than monomeric α-Syn [57].
